# Identification of the Crucial Role of CCL22 in *F. nucleatum*-Related Colorectal Tumorigenesis that Correlates With Tumor Microenvironment and Immune Checkpoint Therapy

**DOI:** 10.3389/fgene.2022.811900

**Published:** 2022-02-28

**Authors:** Hufei Wang, Kangjia Luo, Zilong Guan, Zhi Li, Jun Xiang, Suwen Ou, Yangbao Tao, Songlin Ran, Jinhua Ye, Tianyi Ma, Tianyu Qiao, Zhiming Zhang, Yinghu Jin, Yanni Song, Rui Huang

**Affiliations:** ^1^ Department of Colorectal Cancer Surgery, The Second Affiliated Hospital of Harbin Medical University, Harbin, China; ^2^ Department of Obstetrics and Gynecology, Second Affiliated Hospital of Harbin Medical University, Harbin, China; ^3^ Department of Thoracic Surgery, Second Affiliated Hospital of Harbin Medical University, Harbin, China; ^4^ Department of Breast Surgery, The Third Affiliated Hospital of Harbin Medical University, Harbin, China

**Keywords:** *Fusobacterium nucleatum*, tumor microenvironment, immune checkpoint therapy, chemokines, CCL22

## Abstract

Colorectal cancer (CRC) is the third most common malignant cancer worldwide with the second highest mortality. Gut microbiota can educate the tumor microenvironment (TME), consequently influencing the efficacy of immune checkpoint inhibitors (ICIs). Fusobacterium *nucleatum* is one of the most crucial bacteria contributing to colorectal tumorigenesis, but the molecular mechanisms between *F. nucleatum* and TME or ICIs are poorly investigated. In the present study, we firstly analyzed differentially expressed genes and the biological functions between *F. nucleatum*-infected and uninfected CRC cell lines, with the findings that CCL22 mRNA expression was markedly upregulated after *F. nucleatum* infection. Moreover, the survival analysis showed that CCL22 was significantly associated with the overall survival of CRC patients. Gene Ontology and Kyoto Encyclopedia of Genes and Genomes analysis suggested that CCL22 was related to immune-related terms. Furthermore, the ESTIMATE analysis indicated that the high-CCL22-expression subgroup had a higher immune/stromal/estimate score and lower tumor purity. The CIBERSORT analysis indicated that the high-CCL22-expression group had more immune-suppressive cells and less antitumor immune cells. In addition, immune checkpoint genes and cytotoxic genes were positively correlated with CCL22 expression. The immunophenoscore analysis suggested that CCL22 was associated with the IPS-CTLA4 and PD1/PD-L1/PD-L2 score. Interestingly, CCL22 expression in the KRAS and APC mutation groups was markedly reduced compared to that of the wild groups. In summary, our study provided evidence that CCL22 might play a crucial role in *F. nucleatum*-related colorectal tumorigenesis and correlate with TME and ICIs, which deserves further study.

## Introduction

Colorectal cancer (CRC) is the third most common malignant cancer worldwide with the second highest mortality (Siegel et al., 2020a; Siegel et al., 2020b). In order to seek effective treatment options, it is urgent to find new therapeutic targets of CRC.

*Fusobacterium nucleatum* (*F. nucleatum*), a common member of the oral microbiota known to have a symbiotic relationship with its hosts, has been shown to play a critical role in the development of CRC. Studies have reported that *F. nucleatum* influenced cell metastasis, proliferation, and migration mediated by abnormalities of lncRNA expression, activation of autophagy, and alteration of metabolism (Yang et al., 2017; Yu et al., 2017; Hong et al., 2020). Importantly, *F. nucleatum* can influence colorectal tumorigenesis directly by regulating the tumor microenvironment (TME) *via* increasing myeloid-derived suppressor cells, inhibiting the receptors of natural killer (NK) cells, and controlling T-cell-mediated immune responses (Kostic et al., 2013; Mima et al., 2016; Dong et al., 2020; Serna et al., 2020), which significantly affected the therapeutic response and clinical outcome of patients (Quail and Joyce, 2013; Wood et al., 2014). Hence, dissecting the underlying mechanism of cross-talk between *F. nucleatum* and TME will help search for the potential therapeutic targets.

TME, mainly constituting immune cells and stromal cells (Guo et al., 2018; Stankovic et al., 2018), can shape the development of tumor and impact the response to tumor therapy (Böttcher et al., 2018). Regulating TME is one of the most promising strategies for tumor therapy—for instance, more CD8^+^ T cell infiltration in CRC was generally associated with a favorable prognosis (Galon et al., 2006; Nazemalhosseini-Mojarad et al., 2019). In addition, studies have shown that TME also influenced the response to immune checkpoint inhibitors (ICIs). Christopher *et al*. found that successful anti-PD-1 immunotherapy required the communication of T cells and dendritic cells, which involves the cytokines (Garris et al., 2018). Another study revealed that the efficacy of anti-CTLA-4 therapy relied on regulatory T cell (Treg) depletion during treatment (Arce Vargas et al., 2018). Yu *et al*. also pointed out that re-modulating the TME enhanced the effect of anti-PD-1 immunotherapy in CRC patients with microsatellite stability (Yu et al., 2019). However, the specific molecular mechanisms underlying the interactions between *F. nucleatum* and the TME or ICIs are poorly understood.

Chemokines, a family of low-molecular-weight proteins, are important parts of the communication of tumor cells and the TME and involved in shaping the immune system in modulating immune cell infiltration (Cabrero-de Las Heras and Martínez-Balibrea, 2018). As a member of the chemokine family, CCL22 was reported to promote Treg communication with dendritic cells to control immunity through their CCR4 receptor in lymph nodes (Rapp et al., 2019). CCL22 also promoted Treg recruitment into the TME and inhibited anticancer immunity in melanoma (Anz et al., 2015; Martinenaite et al., 2016). In addition, some studies showed that CCL22 mRNA expression was significantly higher in tumor tissue compared with paired normal tissue in colorectal adenocarcinomas (Wågsäter et al., 2008; Huang et al., 2015; Heeran et al., 2021). Notably, CCL22 was expressed on exposure to gut microbiota and correlated with Treg and Th1 in CRC (Cremonesi et al., 2018). Wang *et al*. found that the upregulation of CCL22 recruited Th17 cells to promote colon carcinogenesis in miR-34a−/− mice infected by *Citrobacter rodentium* (Wang et al., 2018), which linked the upregulation of CCL22 to the gut microbiota. However, the role of CCL22 in *F. nucleatum*-associated TME has not been well studied.

In this research, we firstly found that the expression of CCL22 was upregulated in CRC cell lines infected by *F. nucleatum* using Gene Expression Omnibus (GEO) datasets. The upregulation of CCL22 was accompanied by an increase in immune score and a decrease in stromal score in patients of The Cancer Genome Atlas (TCGA) colon adenocarcinoma (COAD). A further analysis of the composition of immune cells in the TME showed that the high-CCL22-expression subgroup had more immune-suppressive cells (such as Treg and T follicular helper cells) and less antitumor immune cells (such as activated NK cells). In addition, CCL22 was positively correlated with immune checkpoint genes (BTLA, CTLA4, TIGIT, HAVCR2, CD274, PDCD1, and LAG3) and cytotoxic genes (TNFSF11, GZMA, IFNG, PRF1, GZMK, and GZMM). The IPS-CTLA4 and PD1/PD-L1/PD-L2 score was higher in the high-CCL22-expression subgroup. We also found that the expression of CCL22 was related to overall survival (OS), M stage, APC mutation, and KRAS mutation in TCGA COAD patients. In summary, these results indicated that CCL22 might play a pivotal role in *F. nucleatum*-related colorectal tumorigenesis and correlate with the TME and immune checkpoint therapy.

## Materials and Methods

### Data Source

The RNA expression data of CRC cell lines infected by *F. nucleatum* was downloaded from the GEO database, with accession numbers GEO: GSE141805 (HCT-116) and GSE90944 (HT-29), which respectively contains three pairs of samples (*F. nucleatum vs*. control). In addition, the RNA expression data and clinicopathological information of TCGA COAD were obtained from the TCGA database using the “TCGA-biolinks” package in R Studio (Colaprico et al., 2016). Count value was transformed to transcripts per million (TPM) for further analysis, and GSE39582, with a large sample size and complete survival information, was downloaded as a validation dataset. All data were normalized to ensure standardization. The gene symbols that were detected in more than one probe were kept for further analysis. In total, 20,407 immune-related genes (IRGs) were downloaded from the Molecular Signature Database (MSigDB) C7 immunologic signature gene sets (Subramanian et al., 2005). The detailed information of datasets and online websites used in our study are shown in Table 1 and Table 2.

**TABLE 1:** Detailed information of the datasets used in our study.

Data name	Experiment type	Sample	Usage
GSE141805	High-throughput sequencing	*F. nucleatum*-treated HCT-116 colorectal cancer (CRC) cell lines (3)	Differential analysis
		Normal control HCT-116 CRC cell lines (3)	Gene set variation analysis (GSVA)
GSE90944	High-throughput sequencing	*F. nucleatum*-treated HT-29 CRC cell lines (3)	Differential analysis
		Normal control HT-29 CRC cell lines (3)	GSVA
GSE39582	Array	Survival analysis (536)	Survival analysis
TCGA COAD	High-throughput sequencing	Colon cancer samples (480)	Correlation analysis
			Immunophenoscore analysis
		Survival analysis (453)	Survival analysis
		Mutation analysis (399)	Mutation analysis

**TABLE 2:** Detailed information of the online websites used in our study.

Website name	Version	Usage	Accession
MSigDB	v7.4	To download immune-related genes from C7	http://gsea-msigdb.org/gsea/msigdb/
		To obtain the reference gene set for gene set variation analysis from C2. kegg	
GEPIA	v1.0	To obtain the top 100 genes with expression similar to CCL22	http://gepia.cancer-pku.cn/
TIMER	v2.0	To analyze the relationships between CCL22 expression and KRAS/APC mutation	http://timer.comp-genomics.org/
TCIA	v1.0	To obtain the immunophenoscore of The Cancer Genome Atlas colon adenocarcinoma that predicts the response to immune checkpoint inhibitors	https://tcia.at/

### Differential Gene Analysis

GSE141805 and GSE90944 were used to explore the differentially expressed genes (DEGs) between *F. nucleatum*‐infected and uninfected CRC cell lines *via* the R package “edgeR” in R Studio (Robinson et al., 2010). The cutoff threshold is *p*-value <.05 and |log2FC| ≥ 1. To select the IRGs related to *F. nucleatum* infection, immune-related (IR) DEGs were obtained by intersecting IRGs and DEGs, which were visualized using the “venn” package.

### Gene Set Variation Analysis

Gene set variation analysis (GSVA) was performed to investigate the underlying functions and pathways affected by *F. nucleatum* with the “GSVA” package (Hänzelmann et al., 2013). The gene set “c2.cp.kegg.v6.2.symbols.gmt” in MSigDB was selected as the reference gene set. The heat map of enrichment terms was visualized using the “pheatmap” package.

### K–M Survival Analysis

To further analyze the prognostic power of the IR DEGs, patients of TCGA-COAD and GSE39582 were divided into high-expression and low-expression subgroups based on the median expression value of each differentially expressed (DE) IRG, and a survival analysis was conducted by the Kaplan–Meier (K–M) method using the “survival” package.

### Functional Enrichment Analysis

To explore the potential biological functions related to CCL22, the top 100 genes expressing similarly to CCL22 (Sun et al., 2021), downloaded from GEPIA 1.0 website (Tang et al., 2017), were subjected to Gene Ontology (GO) and Kyoto Encyclopedia of Genes and Genomes (KEGG) analysis using the “clusterProfiler” package (Yu et al., 2012), which was also used for the visualization of enrichment terms. In addition, Gene Set Enrichment Analysis (GSEA) was applied to explore the changed biological functions based on the high- and low-CCL22-expression subgroups in TCGA COAD using the “clusterProfiler” package. The GSEA results of GO and KEGG were respectively calculated based on MSigDB c5.all.v7.0.symbols.gmt and c2.cp.kegg.v7.0.symbols.gmt in R studio, which was visualized using the “enrichplot” package.

### Evaluation of Tumor Microenvironment

To dissect the TME associated with CCL22, TCGA-COAD TPM was used to calculate the estimate/immune/stromal score and tumor purity using the “estimate” package based on the high- and low-CCL22-expression subgroups (Yoshihara et al., 2013). Then, the “CIBERSORT” package was used to estimate the proportions of 22 types of immune cells in the TME (Newman et al., 2015).

### Immunophenoscore Analysis

Immunophenoscore (IPS), calculated based on the four main types of genes that determine immunogenicity, has the ability to predict the patients’ response to ICIs (Charoentong et al., 2017). The IPS range is between 0 and 10. The higher the score, the stronger the immunogenicity and the better the response to ICIs. The IPSs of TCGA COAD patients were downloaded from The Cancer Immunome Atlas (TCIA) (Charoentong et al., 2017).

### Mutation Analysis

The mutation data of 399 COAD patients were obtained from the TCGA website, which was analyzed using the “maftools” package in R Studio (Mayakonda et al., 2018). The tumor mutation burden (TMB) was calculated using the formula: (total mutation / total covered bases) × 106. Then, TIMER 2.0 website was used to investigate the CCL22 mutation status in COAD and the correlation between APC/KRAS mutation and CCL22 expression (Li et al., 2020).

### Statistical Analysis

All statistical analyses were performed in R 4.0.3 and its appropriate packages. Data were analyzed with standard statistical tests as appropriate. * represented *p* <.05, ** represented *p* <.01, *** represented *p* <.001, and ns represented no statistical difference.

## Results

### *F. nucleatum* Affects Gene Expression and Biological Functions in Colorectal Cancer Cells

**FIGURE 1:**
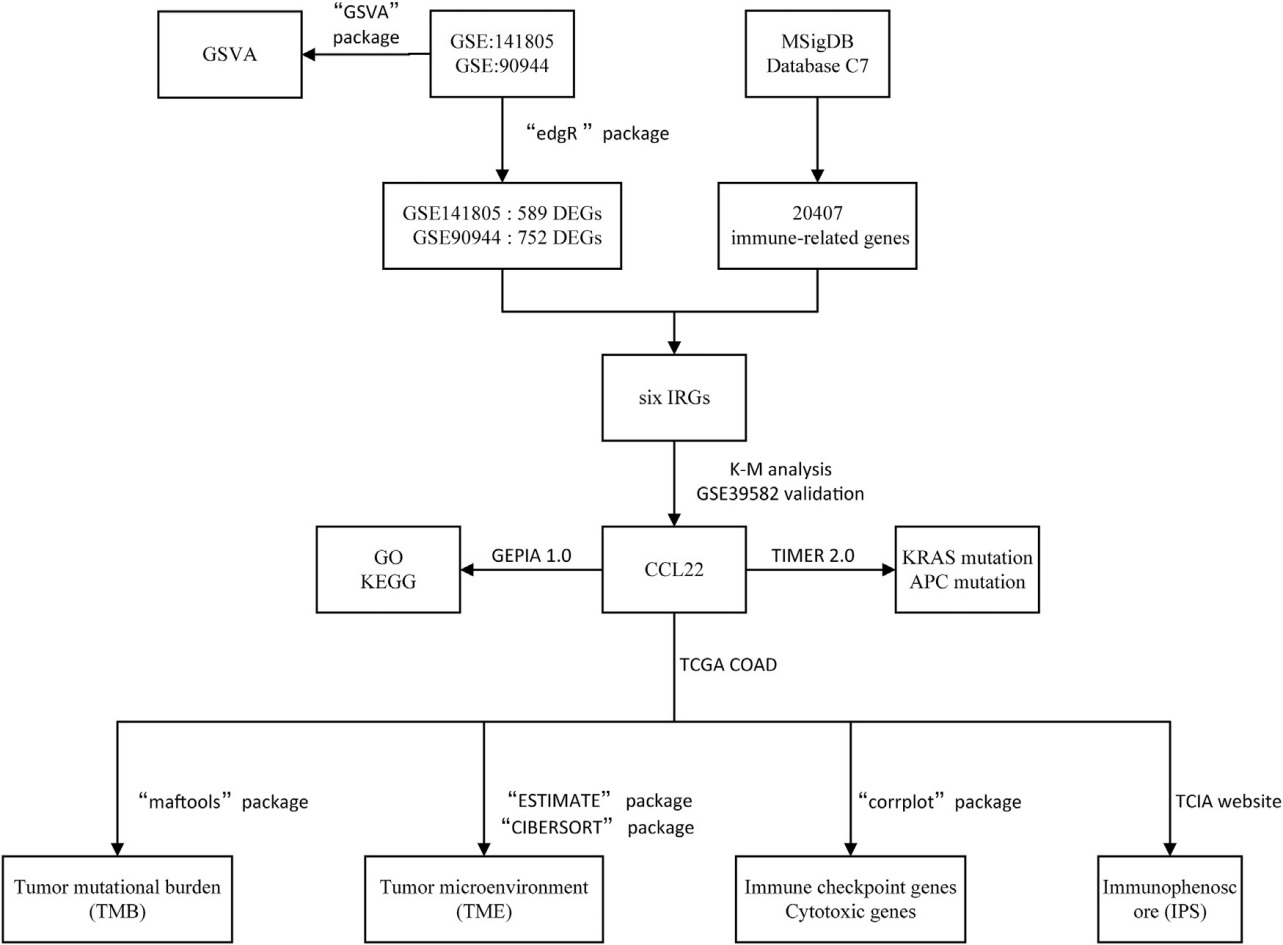
Flow diagram of the present study.

**FIGURE 2:**
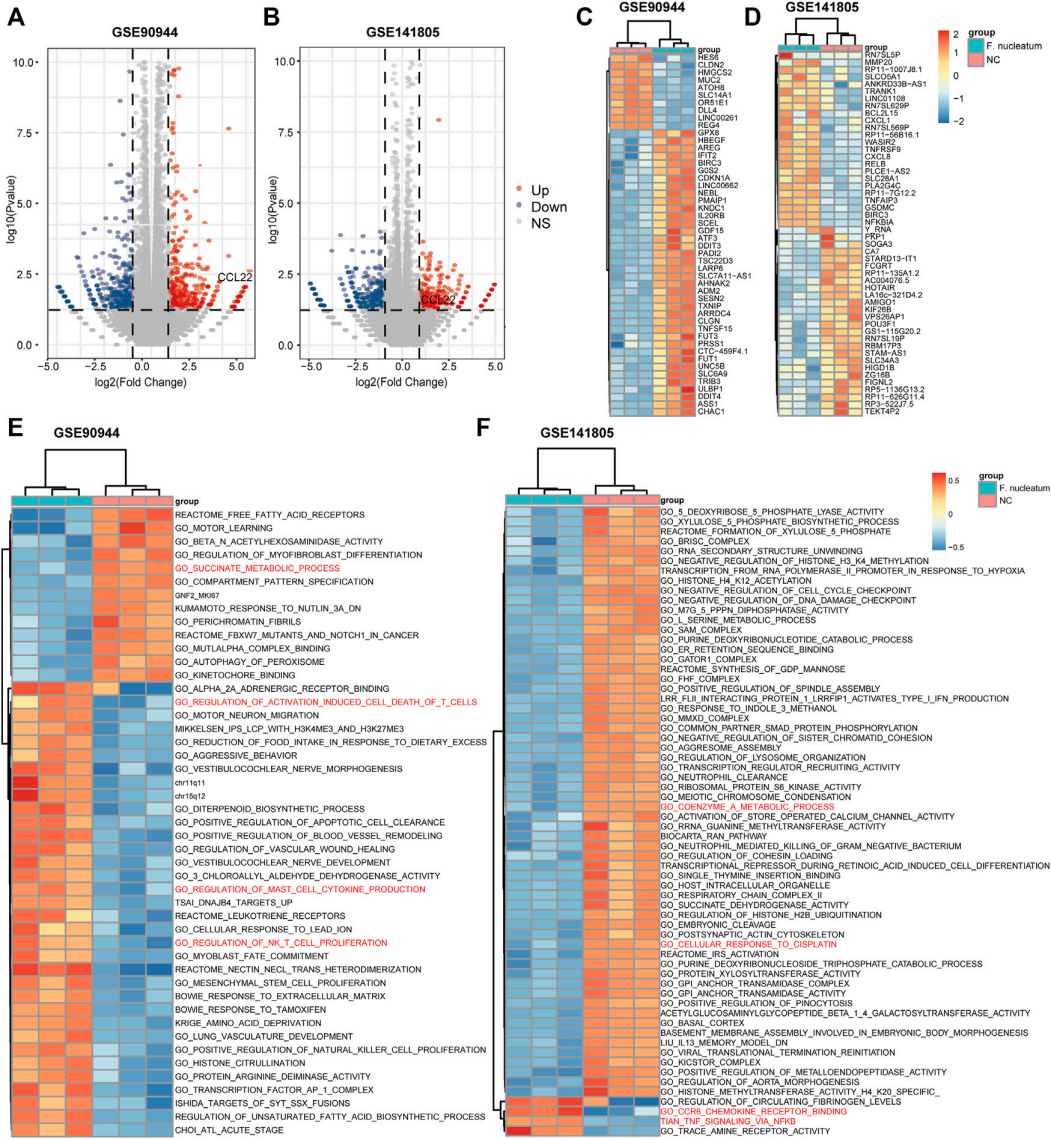
Differentially expressed genes (DEGs) and biological functions affected by *F. nucleatum*. **(A**, **B)** Volcano plots of DEGs influenced by *F. nucleatum* infection. Red dots represent upregulated genes, while blue dots represent downregulated genes. DEGs were selected based on *p*-value <.05 and |log2FC| ≥ 1. Heat maps of the top 50 DEGs **(C**, **D)** and biological functions **(E**, **F)** between *F. nucleatum*-infected and uninfected colorectal cancer cells.

The flow diagram of our study is shown in Figure 1. Based on *p*-value <.05 and |log2FC| ≥ 1.0, we respectively obtained 752 DEGs (373 upregulated and 379 downregulated) in GSE90944 and 589 DEGs (260 up upregulated and 329 downregulated) in GSE141805, which were displayed as volcano plots (Figures 2A,B). The top 50 DEGs were visualized using heat maps (Figures 2C,D). The detailed information of the DEGs is shown in Supplementary Table S1. GSVA was used to further investigate the biological function affected by *F. nucleatum* infection. The biological functions were visualized according to *p*-value <.05 and |log2FC| ≥ 1.0 (Figures 2E,F; Supplementary Table S2). Surprisingly, there were some biological functions that we were interested in. As shown in Figure 2E, we found that “GO_SUCCINATE_METABOLIC_PROCESS” was downregulated and “GO_REGULATION_OF_ACTIVATION_INDUCED_CELL_DEATH_OF_T_CELLS”, “GO_REGULATION_OF_MAST_CELL_CYTOKINE_PRODUCTION”, and “GO_REGULATION_OF_NK_T_CELL_PROLIFERATION” were upregulated in GSE90944. In GSE141805 (Figure 2F), “TIAN_TNF_SIGNALING_VIA_NFKB” and “GO_CCR6_CHEMOKINE_RECEPTOR_BINDING” were upregulated, and “GO_COENZYME_A_METABOLIC_PROCESS” and “GO_CELLULAR_RESPONSE_TO_CISPLATIN” were downregulated. The enrichment biological functions highlighted the crucial role of tumor immunity in *F. nucleatum*-related colorectal cancer.

### CCL22 Plays a Crucial Role in Colorectal Cancer Cells Infected by *F. nucleatum*

**FIGURE 3:**
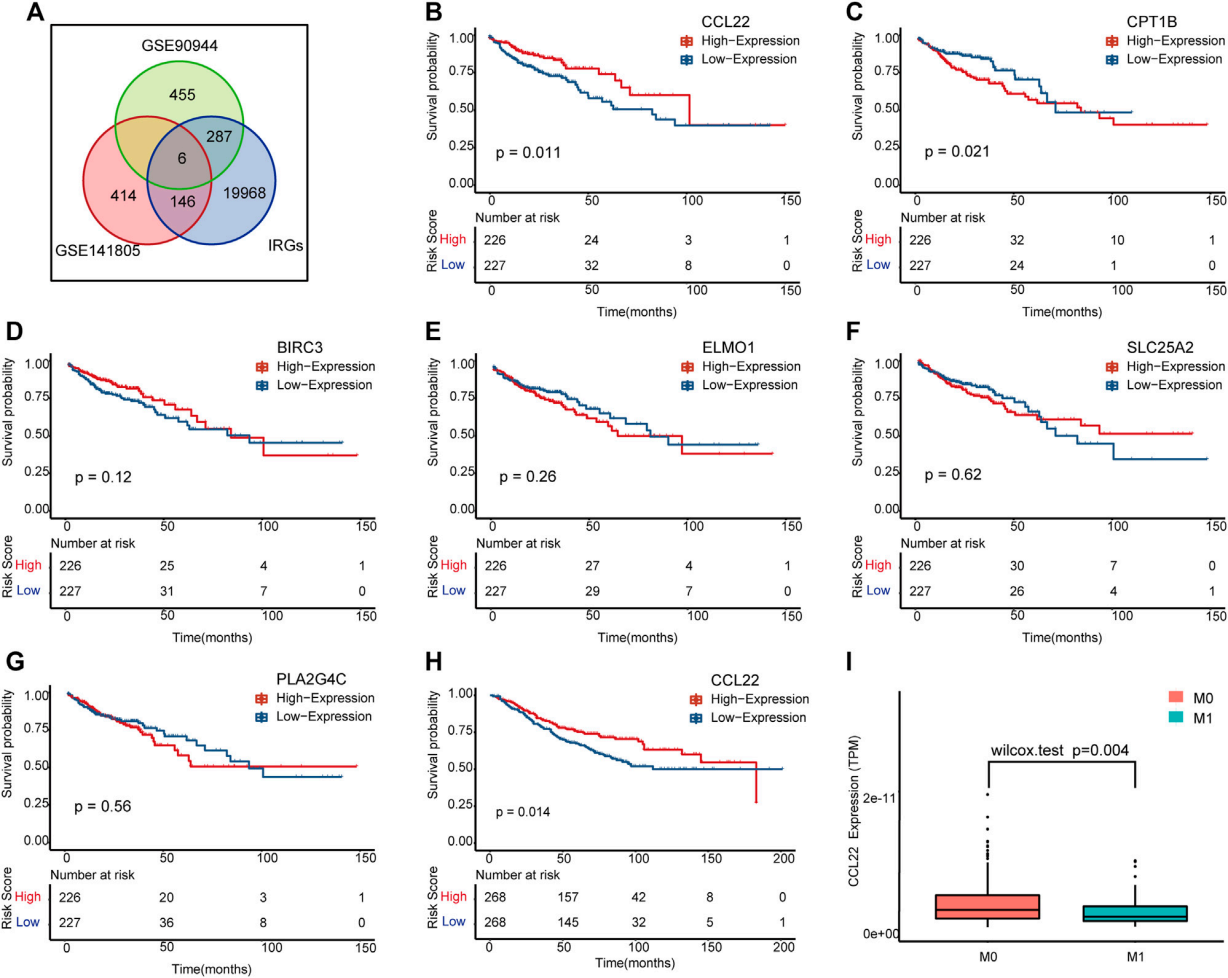
The meaningful immune-related (IR) differentially expressed genes (DEGs) influenced by *F. nucleatum*. **(A)** Venn diagram showing the common genes in GSE90944, GSE141805, and immune-related genes. **(B**–**G)** Kaplan–Meier survival plots of the IR DEGs in The Cancer Genome Atlas (TCGA) colon adenocarcinoma (COAD). **(H)** Kaplan–Meier survival plot of CCL22 in GSE39582. **(I)** Box plot of CCL22 expression between M0 and M1 stages in TCGA COAD.

To explore the relationship between *F. nucleatum* and immunity, we intersected DEGs of the two datasets with IRGs, and a total of six genes remained, including BIRC3, CCL22, CPT1B, ELMO1, PLA2G4C, and SLC25A2 (Figure 3A). Among them, CCL22, BIRC3A, and CPT1B were significantly upregulated under *F. nucleatum* treatment in two datasets. To further determine the meaningful genes, we performed K–M analysis according to the high- and low-expression subgroups of each DE IRG in TCGA (Figures 3B–G) and GSE90944 (Supplementary Figure S1). Interestingly, CCL22 (*p* = .011) and CTP1B (*p* = .021) were selected as meaningful genes in patients of TCGA COAD (Figures 3B,C), but only CCL22 was validated in GSE39582 (Figure 3H), with *p* = .014. CCL22 was differentially expressed in the subgroup analyses according to tumor M stage in TCGA COAD (Figure 3I). By reviewing the literatures, we found that the upregulation of CCL22 played an important role in bacterial and viral infection-associated tumors (Yang et al., 2012; Wang et al., 2018). Hence, we identified CCL22 for further study.

### CCL22 Associated With Immune-Related Biological Functions

**FIGURE 4:**
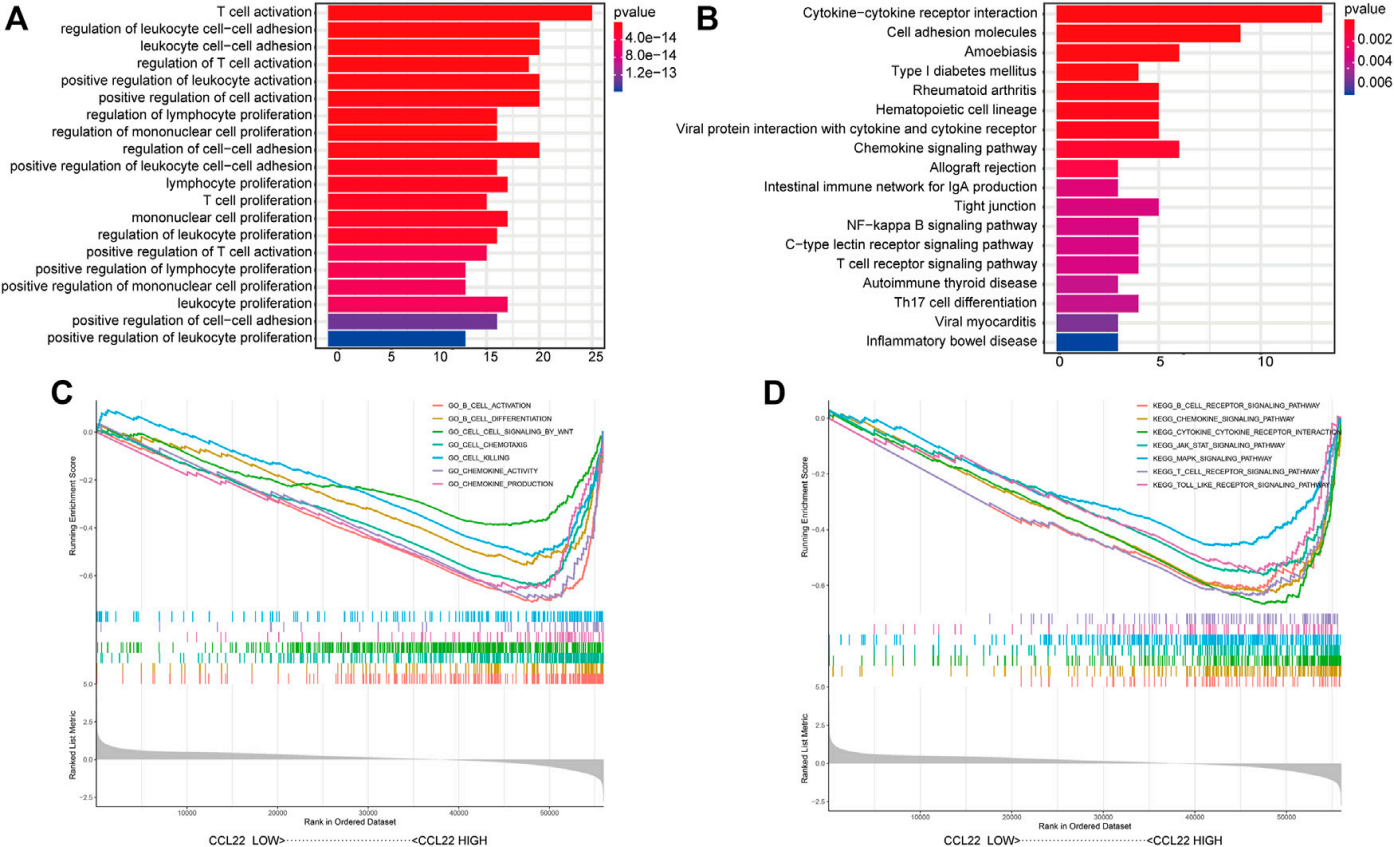
Biological function analysis of CCL22. Representative Gene Ontology (GO) functions **(A)** and Kyoto Encyclopedia of Genes and Genomes (KEGG) pathways **(B)** of the top 100 genes with expression similar to CCL22. Representative Gene Set Enrichment Analysis results of GO functions **(C)** and KEGG pathways **(D)** based on MSigDB.

To dissect the biological functions of CCL22, we identified the top 100 associated genes of CCL22 in COAD using the GEPIA database, followed by KEGG pathway enrichment analysis and GO functional enrichment analysis (Supplementary Table S3). As shown in Figure 4A, the significantly enriched GO (ALL) terms included “T cell activation”, “regulation of leukocyte cell−cell adhesion”, and “lymphocyte proliferation”, which indicated the role of CCL22 in regulating the immune function. The detailed information of GO (BP, CC, and MF) terms are shown in Supplementary Figure S2. The enriched KEGG pathways were as follows: “cytokine–cytokine receptor interaction”, “chemokine signaling pathway”, “intestinal immune network for IgA production”, and “inflammatory bowel disease”, indicating the potential role of CCL22 in gastrointestinal diseases (Figure 4B). Interestingly, we also found that the “NF-kappa B signaling pathway” was related to CCL22, which suggested a potential relationship among NF-kappa B signaling pathway, *F. nucleatum*, and CCL22. In addition, GSEA revealed that GO functions, such as cell–cell signaling by WNT, B cell differentiation, and chemokine production, were markedly enriched in the high-CCL22-expression subgroup (Figure 4C). As for the KEGG pathways, GSEA showed that B cell receptor, chemokine, JAK STAT, and the MAPK signaling pathway were enriched in the high-CCL22-expression subgroup (Figure 4D; Supplementary Table S4).

### CCL22 Modulates the Tumor Microenvironment in TCGA COAD

**FIGURE 5:**
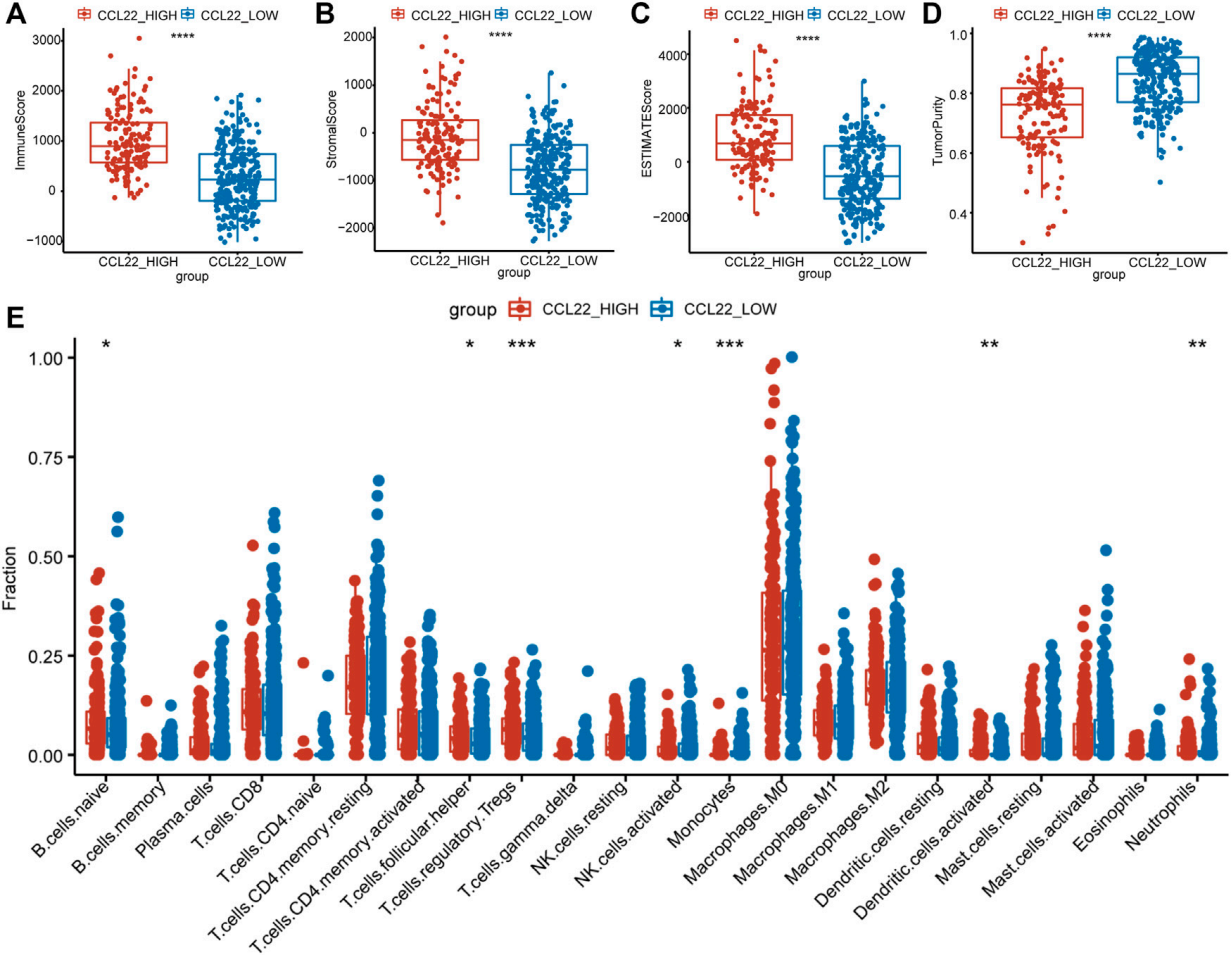
Tumor microenvironment changes associated with CCL22 in The Cancer Genome Atlas colon adenocarcinoma. **(A)** The immune score, **(B)** stromal score, **(C)** estimated score, and **(D)** tumor purity and **(E)** the proportion of 22 types of infiltrating immune cells in high- and low-CCL22-expression subgroups.

Studies have shown that chemokines recruited immune cells into tumor beds and influenced the TME (Marques et al., 2019). To explore whether CCL22 regulated the TME of COAD, ESTIMATE analysis was used to calculate the immune/stromal/estimate score and tumor purity of each patient. We excitedly found that the immune/stromal/estimate scores (Figures 5A–C) were significantly higher, while tumor purity (Figure 5D) was significantly lower in the high-CCL22-expression subgroup compared to the low-CCL22-expression subgroup, which suggested that CCL22 was closely related to TME. Then, TPM value was applied to the CIBERSORT algorithm to further determine the composition of immune cells in TCGA COAD tissues with a different CCL22 expression status. We found that CCL22 was positively associated with Treg (*p* < .001), naive B cells (*p* < .05), activated dendritic cells (*p* < .01), neutrophils (*p* < .01), and T follicular helper cell (*p* < .05), while it was negatively associated with activated NK cells (*p* < .05) and monocytes (*p* < .001) in COAD, as shown in Figure 5E.

### CCL22 Related to Immune Checkpoint Therapy

**FIGURE 6:**
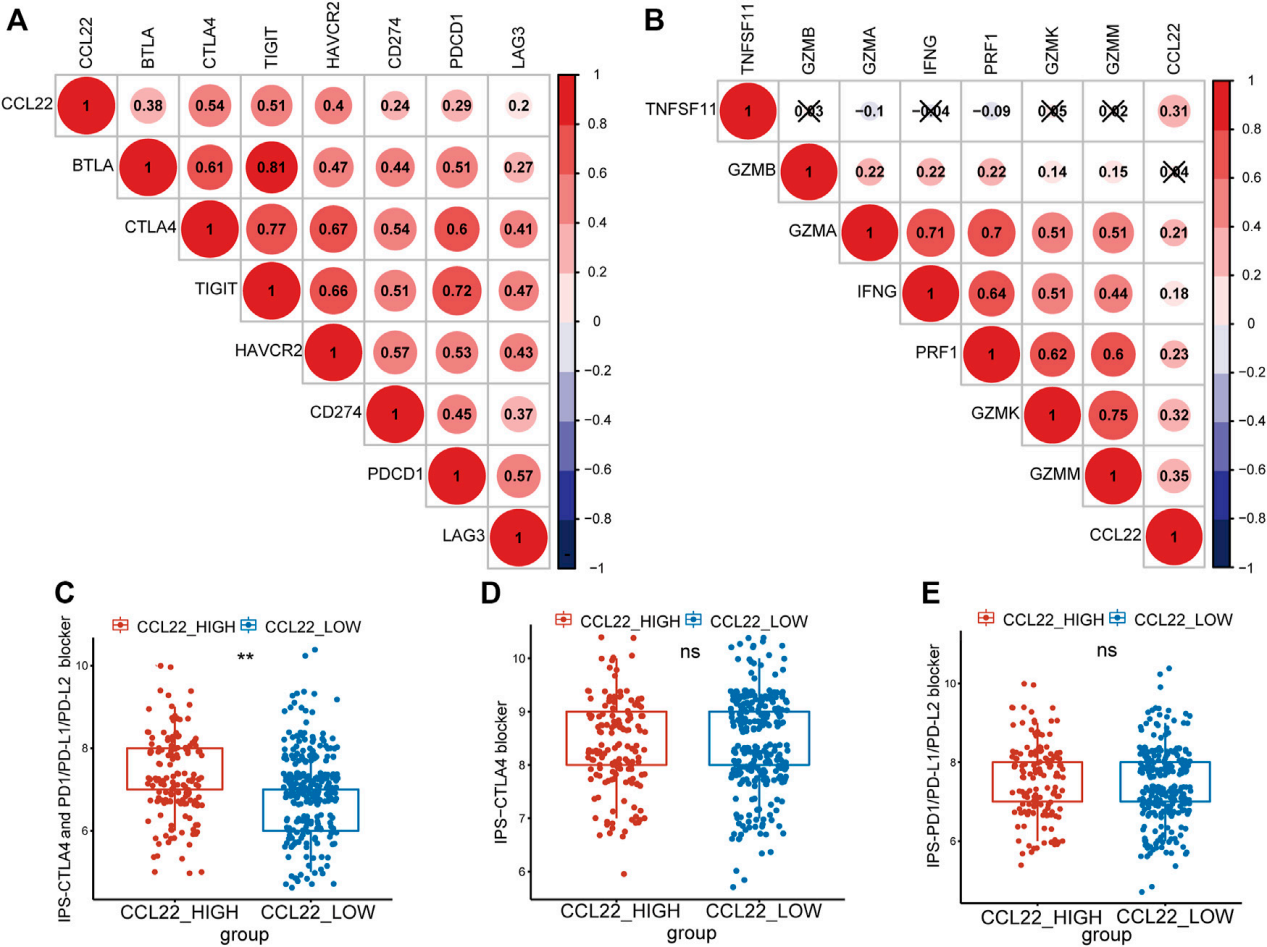
Relationship between CCL22 expression and response to immune checkpoint inhibitors. **(A)** The correlation between CCL22 expression and immune checkpoint genes and **(B)** cytotoxic genes. Red is positive, and blue is negative. The symbol “x” represented a *p*-value >.05, and the circles without “x” meant *p*-value <.05. The numbers in the circle represented the correlation value. Box plots showing the association between **(C)** IPS-CTLA4 and PD1/PD-L1/PD-L2, **(D)** IPS-CTLA4, and **(E)** IPS-PD1/PD-L1/PD-L2 scores and CCL22 expression in patients of The Cancer Genome Atlas colon adenocarcinoma.

Studies have shown that the TME is closely related to the efficacy of immune checkpoint therapy (Tumeh et al., 2014; Hegde et al., 2016). To explore whether CCL22 was also associated with immune checkpoint therapy in COAD, the correlations between CCL22 and immune checkpoint molecules were analyzed (Kim et al., 2017; Nishino et al., 2017; Zhai et al., 2018). As shown in Figure 6A, we found that CCL22 was positively related to BTLA, CTLA4, TIGIT, HAVCR2, CD274, PDCD1, and LAG3 (correlation value = 0.38, 0.54, 0.51, 0.4, 0.24, 0.29, and 0.2; all *p*-value <.05). As shown in Figure 6B, CCL22 expression was positively correlated with cytotoxic genes, such as TNFSF11, GZMA, IFNG, PRF1, GZMK, and GZMM (correlation value = 0.31, 0.21, 0.18, 0.23, 0.32, and 0.35; all *p*-value <.05). It has been reported that IPS is a predictor of response to ICIs based on the TCGA data. To explore the relationship between CCL22 and IPS, the IPS of TCGA COAD was downloaded from the TCIA website. Although there was no statistical difference in IPS-PD1/PD-L1/PD-L2 score and IPS-CTLA4 score between the high- and low-CCL22-expression subgroups (Figures 6D,E), the high-CCL22-expression subgroup had a statistically higher IPS-CTLA4 and PD1/PD-L1/PD-L2 score (Figure 6C).

### CCL22 Associated With APC and KRAS Mutation in TCGA COAD

**FIGURE 7:**
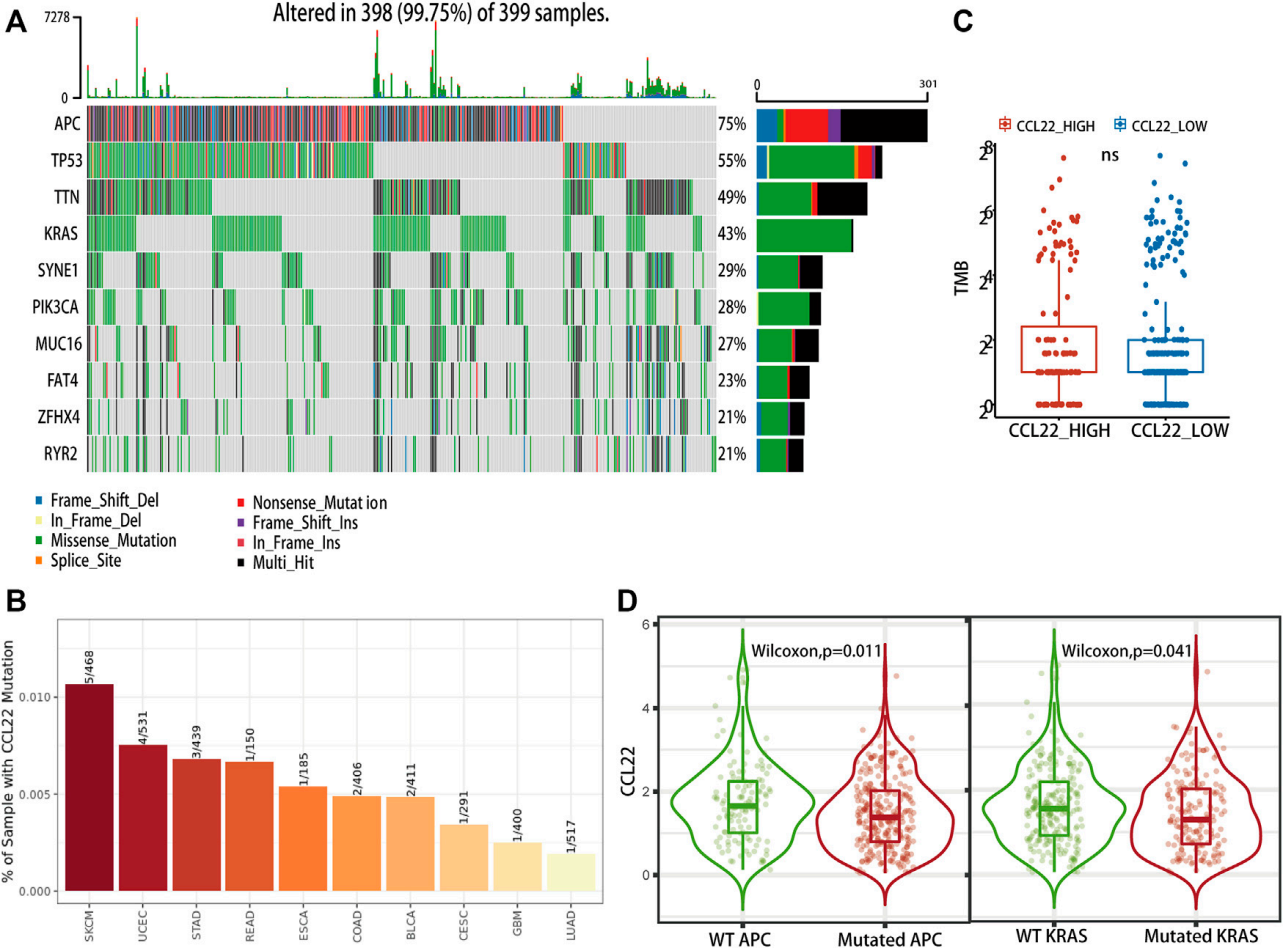
Mutation landscape related to CCL22 in The Cancer Genome Atlas (TCGA) colon adenocarcinoma (COAD). **(A)** Oncoplot showing the top 10 mutational genes in TCGA COAD). **(B)** Mutation status of CCL22 in different cancer types. **(C)** Relationship between CCL22 expression and tumor mutation burden in TCGA COAD. **(D)** Violin plots showing the CCL22 expression in mutant and wild groups of APC and KRAS.

Firstly, we explored the mutation status of CCL22 using the Gene_Mutation module of TIMER 2.0 website. As shown in Figure 7B, only 2 of the 406 samples had CCL22 mutation in COAD, much lower than APC mutation (286 of the 406 samples) and KRAS mutation (174 of the 406 samples), which are shown in Supplementary Figure S3. The top four mutation genes in COAD were APC, TP53, TTN, and KRAS (Figure 7A). The mutation of the above-mentioned genes was closely related to colorectal tumorigenesis. Although there was no difference in TMB between the high- and low-CCL22-expression subgroups (Figure 7C), we were surprised to find that the expression of CCL22 was significantly decreased in the APC and KRAS mutation groups in the Gene_Mutation module of TIMER2.0 website (Figure 7D). This strongly supported the idea that CCL22 might play an important role in colorectal tumorigenesis.

## Discussion

Mounting evidence supports that gut microbiota has a profound influence on the effectiveness of tumor immunotherapy (Vétizou et al., 2015; Gopalakrishnan et al., 2018)—for example, *Enterococcus hirae* and *Barnesiella intestinihominis* enhance cyclophosphamide-induced therapeutic immunomodulatory effects (Daillère et al., 2016). Besides this, *Akkermansia muciniphila* can specifically facilitate the effect of PD-1-based immunotherapy by recruiting T lymphocytes into the tumor beds (Routy et al., 2018), thus suggesting the enormous potential of gut microbiota in regulating the TME and influencing antitumor immune. As one of the most crucial bacteria related to CRC, *F. nucleatum* can influence colorectal tumorigenesis directly by increasing myeloid-derived suppressor cells, inhibiting receptors of NK cells, and controlling T-cell-mediated immune responses (Kostic et al., 2013; Mima et al., 2016; Dong et al., 2020; Serna et al., 2020). The molecular mechanisms underlying the interactions between *F. nucleatum* and the TME or immunotherapy are deemed to be further investigated.

In the present research, we firstly analyzed the gene expression and biological functions between *F. nucleatum*-infected and uninfected CRC cell lines. In total, 589 and 752 DEGs were respectively obtained in the GSE141805 and GSE90944 datasets. We found that *F. nucleatum* upregulated “TIAN_TNF_SIGNALING_VIA_NFKB”. Studies showed that the activation of NF-kappaB induced by *F. nucleatum* participated in metastasis, proliferation, and chemoresistance to 5-fluorouracil (5-Fu) in CRC (Yang et al., 2017; Zhang et al., 2019; Chen et al., 2020). The downregulation of “GO_CELLULAR_RESPONSE_TO_CISPLATIN” suggested that *F. nucleatum* may contribute to chemoresistance to oxaliplatin in CRC, and Hong *et al*. pointed out that *F. nucleatum* promoted carcinogenesis *via* increasing CRC cell glucose metabolism (Hong et al., 2020). According to our analysis, “GO_COENZYME_A_METABOLIC_PROCESS” and “GO_SUCCINATE_METABOLIC_PROCESS” may also be potential mechanisms of colorectal carcinogenesis induced by *F. nucleatum*. What is more, “GO_REGULATION_OF_ACTIVATION_INDUCED_CELL_DEATH_OF_T_CELLS”, “GO_REGULATION_OF_MAST_CELL_CYTOKINE_PRODUCTION”, and “GO_REGULATION_OF_NK_T_CELL_PROLIFERATION” were upregulated. The enrichment biological functions also highlighted the crucial role of tumor immunity in *F. nucleatum*-related CRC, which has been validated in many studies (Kostic et al., 2013; Mima et al., 2015; Hamada et al., 2018).

Modulating tumor immunity was considered to be the most promising treatment for tumor, so we further dissected the effect of *F. nucleatum* on tumor immunity. By intersecting with IRGs, we finally obtained six DE IRGs, including BIRC3, CCL22, CPT1B, ELMO1, PLA2G4C, and SLC25A2. Surprisingly, BIRC3 was reported to upregulate after *F. nucleatum* infection and promote chemoresistance to 5-Fu in CRC (Zhang et al., 2019), which suggested the reliability of our analysis. Furthermore, the survival analysis showed that CCL22 was significantly related to the OS and M stages of CRC patients in TCGA COAD. The prognostic power of CCL22 was also validated in GSE39582. Hence, we identified CCL22 for further study, and GO and KEGG analysis showed that CCL22 were mainly related to immune-related functions. Interestingly, we found that “NF-kappa B signaling pathway” was related to CCL22, which was also induced by *F. nucleatum* infection. This evidence suggested the potential relationships among NF-kappa B signaling pathway, *F. nucleatum*, and CCL22.

It has been reported that CCL22, a member of the chemokine family, can recruit immune cells to rewire the TME *via* binding to CCR4 (Rapp et al., 2019). Our study found that the high-CCL22-expression subgroup had a higher immune/stromal/estimate score and lower tumor purity. The high-CCL22-expression subgroup with a markedly higher immune score suggested more immune cell infiltration. Further analysis of the proportion of various immune cells indicated that the high-CCL22-expression subgroup had more immune-suppressive cells (such as Tregs and T follicular helper cells) and less antitumor immune cells (such as activated NK cells). It seemed that CCL22 induced the immune-suppressive TME to promote colorectal tumorigenesis, and it might be a potential target for *F. nucleatum* to affect the TME.

Immune checkpoint inhibitors show great potential in multiple cancers, such as melanoma, bladder cancer, and prostate cancer (Topalian et al., 2012). However, in CRC, only MSI-H patients (Ganesh et al., 2019), a small proportion of CRC, benefit from ICIs. Our study discovered that the CCL22 mRNA expression was positively correlated with immune checkpoint molecules and cytotoxic genes (Garzón-Tituaña et al., 2020), which were reported to influence the functions of immunocytes (Garzón-Tituaña et al., 2020; Cao et al., 2021). These clues indicated that high-CCL22-expression patients may have a better response to ICIs. It has been reported that IPS was a predictor of response to ICIs based on the TCGA data. A higher IPS score predicted better response to ICIs (Yan et al., 2021). In the present study, we found that the high-CCL22-expression subgroup had statistically higher IPS-CTLA4 and PD1/PD-L1/PD-L2 scores, which also suggested that high-CCL22-expression patients had a better response to the CTLA4 and PD1/PD-L1/PD-L2 combination therapy. Surprisingly, Rapp *et al*. also pointed out that the CCL22–CCR4 axis may serve as an immune checkpoint and was important for inhibiting T cell immunity (Rapp et al., 2019). Klarquist *et al*. found that the vaccination of CCL22 led to redirecting Treg away from tumors, and the repetitive vaccination with CCL22 sufficiently limited Treg accumulation and tumor growth in animals, which carried the potential of local vaccination of CCL22 to enhance the therapeutic effect of ICIs (Klarquist et al., 2016). All of these highlighted the potential role of CCL22 in ICIs.

TMB, defined as the total number of non-synonymous mutations in the coding regions of genes, has been reported as an effective predictor of response to ICIs (Sen et al., 2019). In patients of TCGA COAD, we did not find a statistically different TMB between the high- and low-CCL22-expression subgroups. However, the expression of CCL22 was higher in the KRAS and APC mutation groups compared to the KRAS and APC wild groups. APC and KRAS are the most predominant mutation genes closely associated with colorectal tumorigenesis (Fearon, 2011). In addition, APC mutation was used to construct a spontaneous tumorigenesis mice model of CRC in biological experiments (Moser et al., 1995; Yamada and Mori, 2007), which implied the potential role of CCL22 in colorectal tumorigenesis.

There were still some limitations in our study. On the one hand, experiments *in vitro* and *in vivo* were lacking, and further QPCR and Western blot were needed to verify the expression of CCL22 as well as of other differentially expressed genes (BIRC3, CPT1B, ELMO1, PLA2G4C, and SLC25A2) in CRC cell lines infected by *F. nucleatum*. The effective impact of *F. nucleatum*-induced expression of CCL22 on colorectal tumorigenesis also needed to be validated in the future. On the other hand, the ability of CCL22 and other differentially expressed genes to predict survival needed to be further validated in multicenter clinical samples. Nevertheless, we found that many literatures have suggested the crucial role of CCL22 in *F. nucleatum*-related colorectal tumorigenesis. First, it has been reported that CCL22 expression is elevated in colorectal cancer (Wågsäter et al., 2008; Huang et al., 2015). Gut microbiota infection can induce the expression of some chemokines, including CCL22, in colorectal cancer (Cremonesi et al., 2018). What is more, one study showed that the loss of miR-34a can increase CCL22 expression and promote the development of colorectal cancer after an infection by the bacterium *Citrobacter*, while the NF-KB signaling pathway also plays an important role in the development of colorectal cancer (Wang et al., 2018). Our analysis showed that *F. nucleatum* infection could increase CCL22 expression and influence the NF-KB signaling pathway in two kinds of colorectal cancer cells. Moreover, CCL22 was also related to NF-KB signaling pathway in TCGA-COAD. In summary, it is reasonable to conclude that *F. nucleatum* can also increase CCL22 expression, thereby promoting colorectal tumorigenesis, and the NF-KB signaling pathway is a part of its mechanism.

In summary, our study found that CCL22 mRNA expression was upregulated in CRC cell lines infected by *F. nucleatum*. The upregulation of CCL22 was associated with the TME of COAD, in which the high-CCL22-expression subgroup had more immune-suppressive cells and less antitumor immune cells. The high-CCL22-expression subgroup possessed higher IPS-CTLA4 and PD1/PD-L1/PD-L2 scores. This study provides several supporting lines of evidence that highlight the critical role of CCL22 in *F. nucleatum*-related colorectal tumorigenesis and its close relationship with the TME and ICIs, which deserved further cell and animal experiments.

## Data Availability

The original data presented in the study are included in GEO and TCGA websites (Table 1). Further inquiries can be directed to the corresponding authors.
