# Supplementary material for: Identification of the Crucial Role of CCL22 in F. nucleatum-Related Colorectal Tumorigenesis that Correlates With Tumor Microenvironment and Immune Checkpoint Therapy
Source: Front Genet. 2022 Feb 28;13:811900. doi: 10.3389/fgene.2022.811900 (PMC8918684; doi:10.3389/fgene.2022.811900)
Supplement: Supplementary file 1 [file DataSheet1.docx]

Supplementary Material

Supplementary Figures


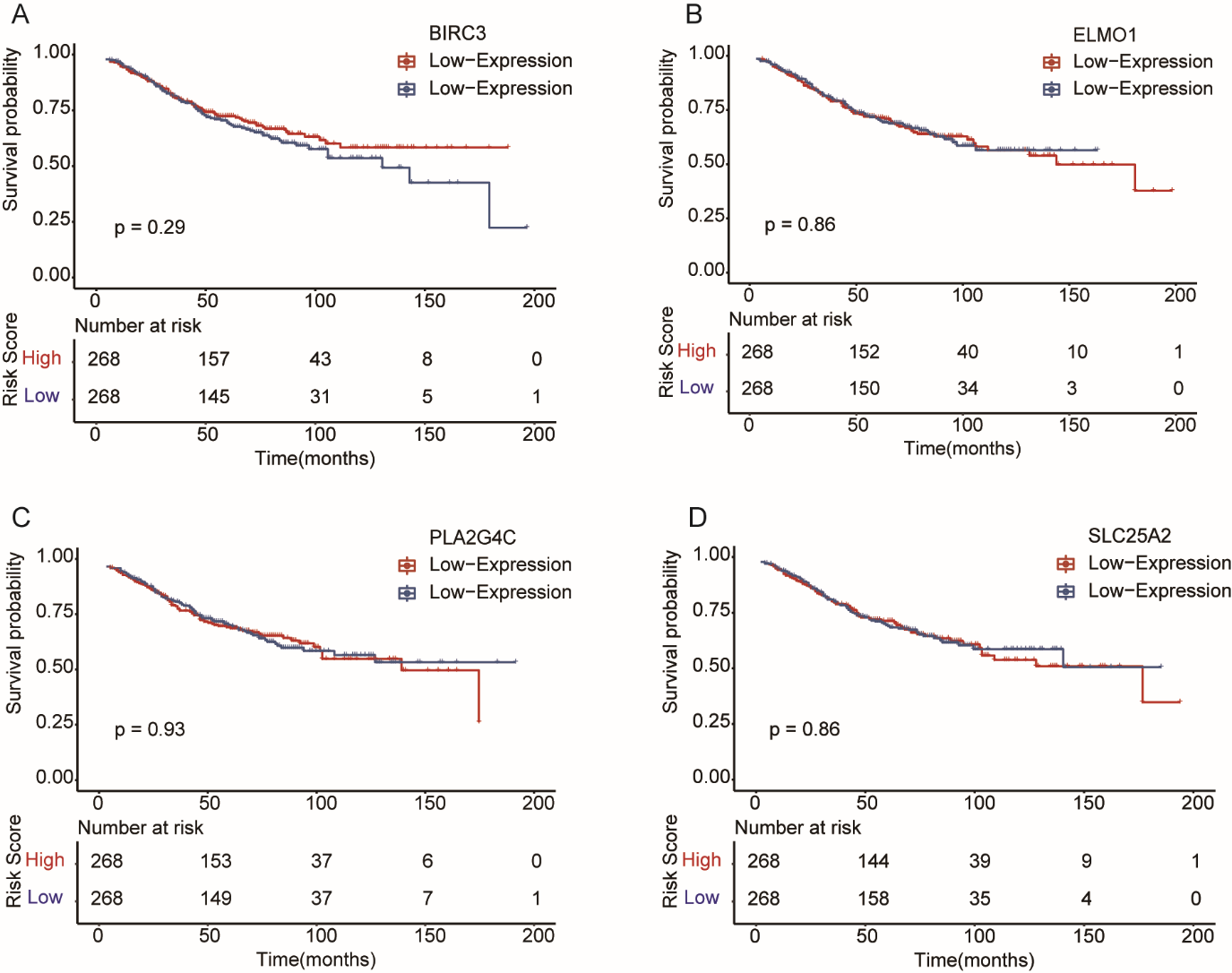


**Supplementary Figure 1.** The Kaplan-Meier survival plots of the other IR DEGs in GSE39582 (A-D). CTP1B was not found in GSE39582.


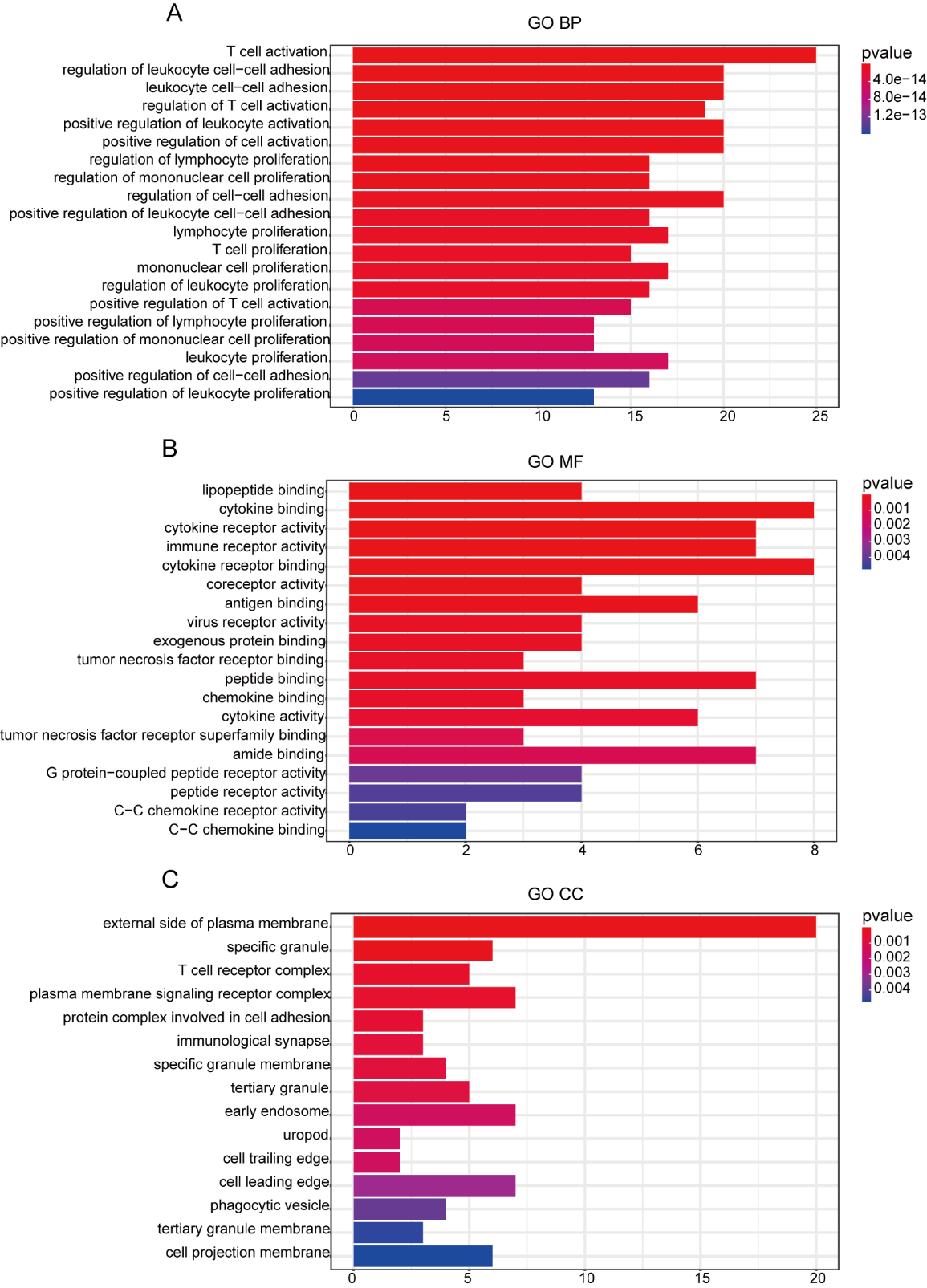


**Supplementary Figure 1.** BP (A), MF (B) and CC (C) terms of top 100 genes expressing similarly to CCL22. BP, Biological Process; MF, Molecular Function; CC, Cellular Component.


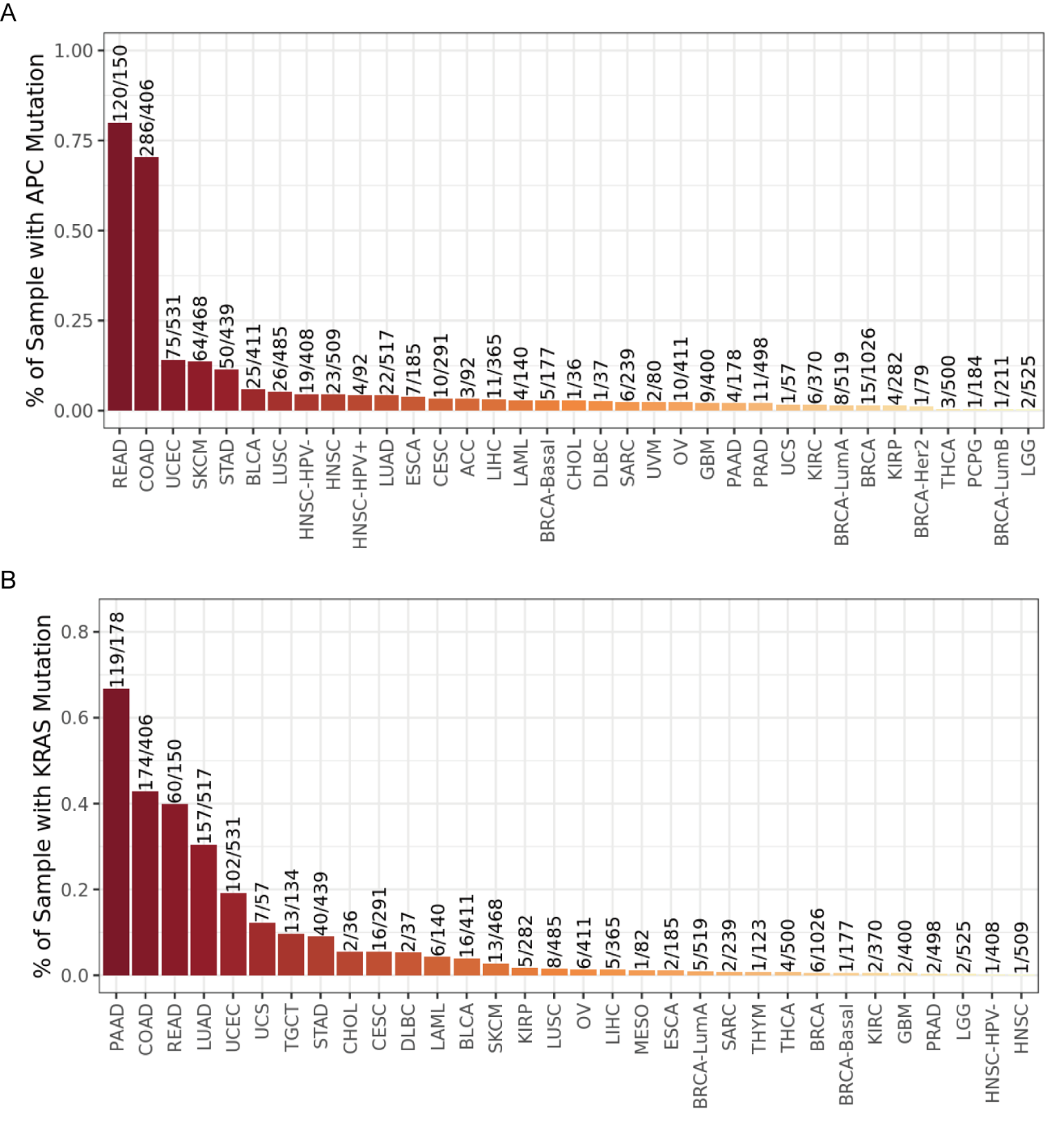


**Supplementary Figure 1.** The mutation status of APC (A) and KRAS (B) in different cancer types.
